# An Unusual Approach to Diagnosing Stump Appendicitis Using Colonoscopy

**DOI:** 10.7759/cureus.76072

**Published:** 2024-12-20

**Authors:** Bhargava K Nelluri, Anupam K Gupta

**Affiliations:** 1 Internal Medicine, SSM (Sisters of Saint Mary) Health Good Samaritan Hospital, Mount Vernon, USA; 2 Surgery, SSM (Sisters of Saint Mary) Health Good Samaritan Hospital, Mount Vernon, USA

**Keywords:** appendicitis, colonoscopy, laparoscopic stump appendectomy, recurrent appendicitis, stump appendectomy, stump appendicitis

## Abstract

Stump appendicitis is a known post-appendectomy entity causing right lower quadrant abdominal pain. Usually, a patient with a prior history of appendectomy presents to the emergency room with right lower quadrant abdominal pain and stump appendicitis, which is visualized on computed tomography of the abdomen pelvis. We report a case of stump appendicitis diagnosed by colonoscopy and subsequently confirmed by surgery.

## Introduction

The vermiform appendix is a vestigial structure at the base of the cecum [1]. Appendicitis is a leading cause of acute abdominal pain, resulting from inflammation of the appendix [2]. Laparoscopic appendectomy for acute appendicitis is a standard surgical procedure [3]. Stump appendicitis is an inflammatory presentation of the remaining appendicular stump and usually presents with abdominal pain and symptoms similar to acute appendicitis [4,5]. Most commonly, an imaging modality helps diagnose this entity. 

We report the case of a 59-year-old female patient presenting with recurrent bouts of right lower quadrant abdominal pain in whom computed tomography (CT) was unable to identify the appendicular stump. A subsequent colonoscopy revealed an appendicular lumen with symptoms mimicking acute appendicitis post colonoscopy, which led to diagnostic laparoscopy. This helped identify a stump appendix that was causing her repeated bouts of abdominal pain.

## Case presentation

**Figure 1:**
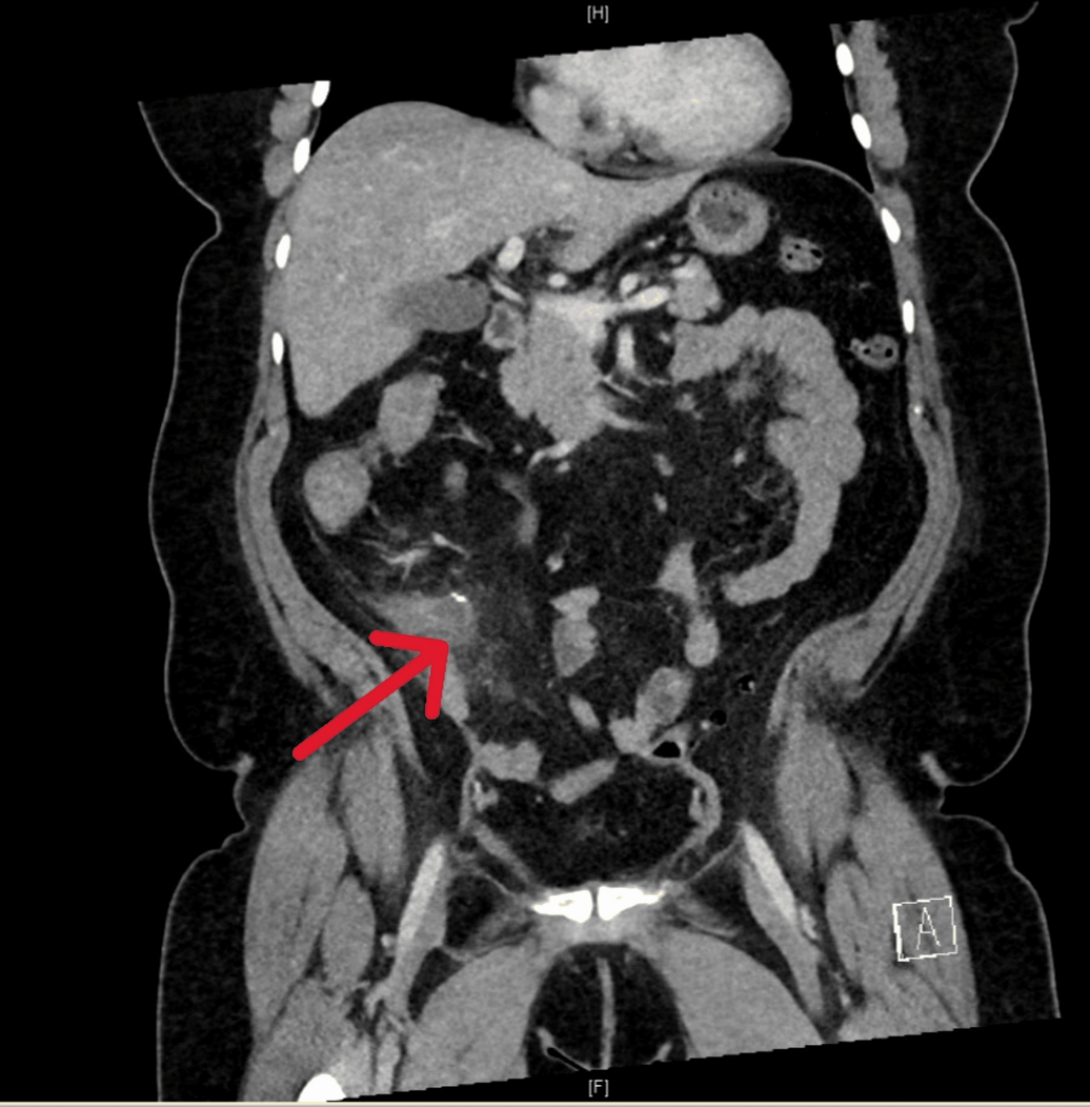
Computed tomography of abdomen and pelvis showing pericaecal image

A 59-year-old female patient with a history of coronary artery disease status post cardiac stenting, hypertension hyperlipidemia, and appendectomy six months earlier, presented to the emergency room with complaints of right lower abdominal quadrant pain, nausea, vomiting, and fever. Her blood work at arrival was normal, other than neutrophilia in her differential count. Her body mass index was 39 kg/m^2^. A CT of the abdomen pelvis revealed evidence of prior appendectomy and inflammatory changes around the cecum (Figure 1). The patient responded to broad-spectrum antibiotics and supportive measures, which led to the resolution of symptoms over the next few days.

**Figure 2:**
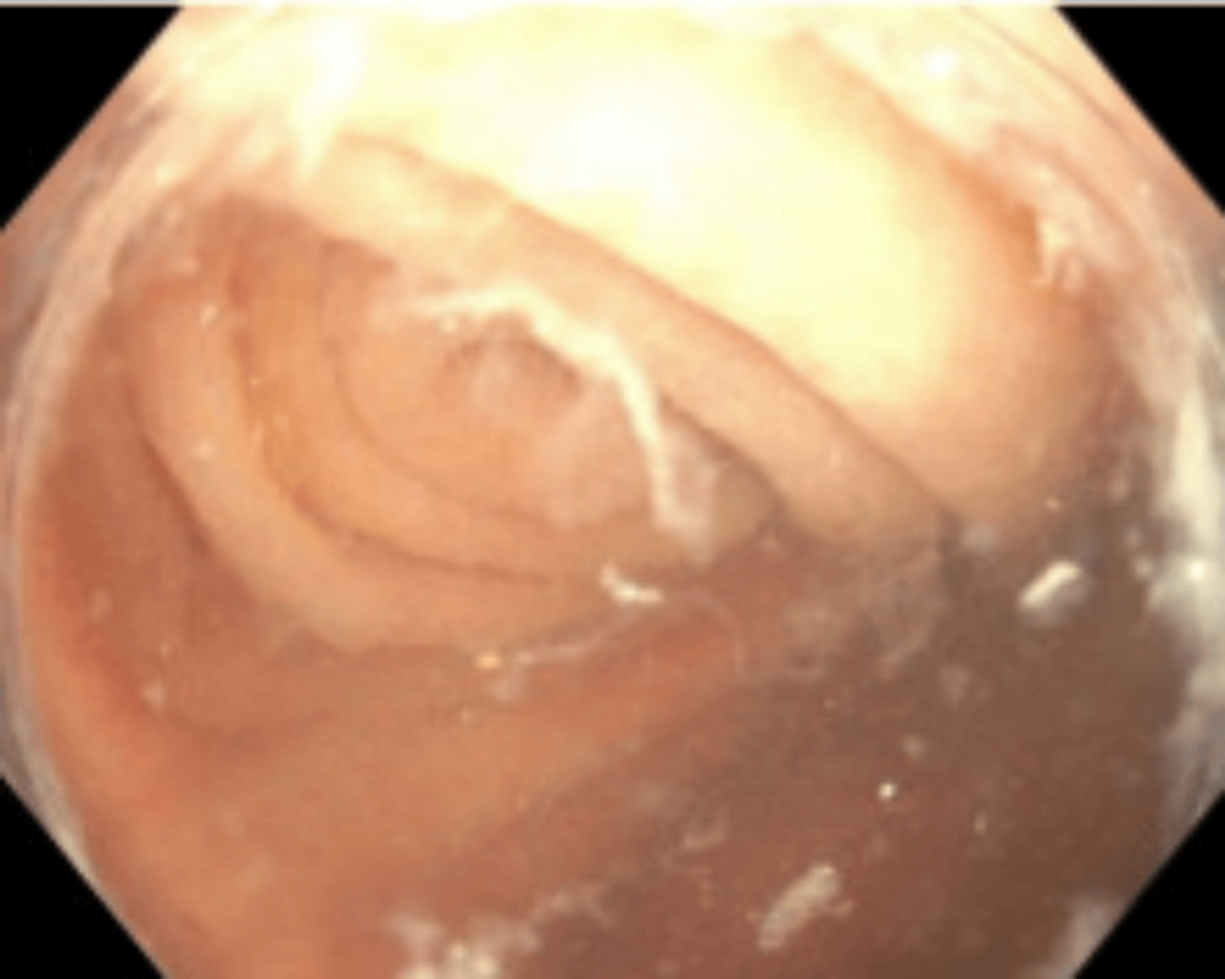
Appendicular area seen on colonoscopy

**Figure 3:**
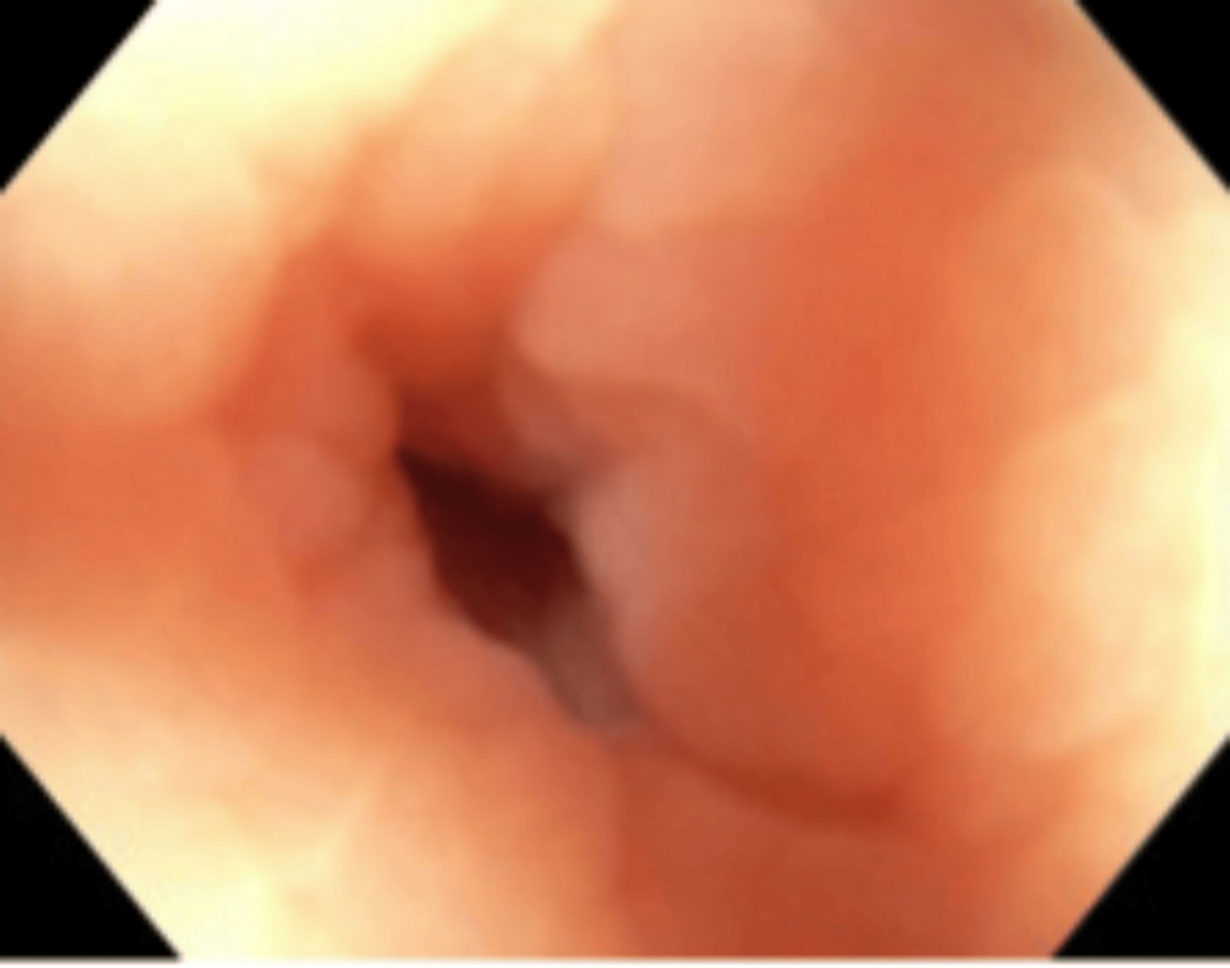
Appendicular lumen seen on colonoscopy

**Figure 4:**
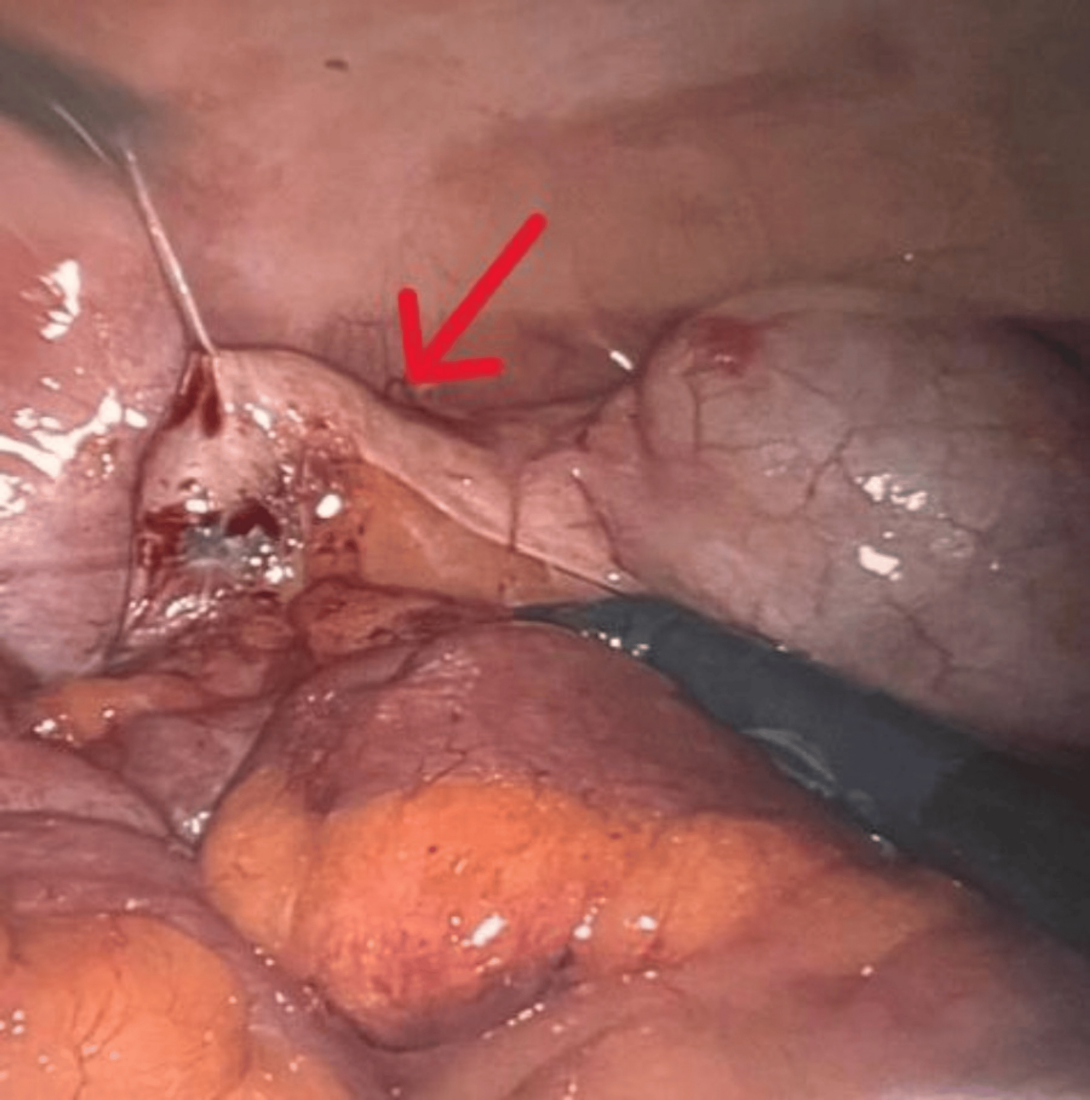
Appendicular stump seen during surgery

Given the lack of clear etiology for the symptoms and her age of 59, the patient underwent a colonoscopy about six weeks after her acute inflammatory episode. During the colonoscopy, an appendicular orifice was identified. Careful evaluation of the appendicular orifice and the cecal area was done to rule out other causes of cecal pathology (Figures 2, 3). Post colonoscopy, the patient had a recurrent similar episode of right lower quadrant abdominal pain. In view of the patent appendicular orifice and no obvious etiology for her symptoms, the patient underwent diagnostic laparoscopic surgery for further evaluation of her symptoms. Intraoperatively, we identified a 3 cm appendicular stump adherent to the right paracolic gutter near the cecum (Figure 4). The patient underwent appendectomy with removal of the appendicular stump.

## Discussion

Acute appendicitis presents as acute onset of right lower quadrant abdominal pain. Pathophysiology most accepted is usually obstruction of the lumen with inflammation or fecolith, which impedes drainage of the appendix. Once diagnosed, surgical appendectomy is a standard modality for the management of acute appendicitis. Appendectomy usually involves the surgeon stapling or ligating the appendix of the cecum and excision of the inflamed appendix. Based on the inflammation, anatomy, or iatrogenic cause, a stump of the appendix may be left behind. This remaining stump can again present with abdominal pain should the lumen get obstructed again. There are multiple case reports of patients presenting with recurrent abdominal pain and diagnosed to have stump appendicitis on imaging, needing secondary surgery. It is not clear what the incidence of stump appendicitis is. However, it has been described that 15-49% of patients with nonoperative management of appendicitis during the initial management present again with recurrent symptoms [6-9].

In patients over the age of 40 presenting with acute appendicitis status, an increased incidence of right-sided colonic cancer has been reported [10]. Our patient, given her age over 40, underwent a colonoscopy, and a careful visualization of the cecal area, appendicular orifice, and lumen was performed to look for any pathology.

There has been reported evidence of appendicitis post-colonoscopy [11,12]. In our patient, we did not have symptoms strong enough to go back to the emergency room but the patient reported right lower quadrant tenderness post colonoscopy in her subsequent visit to the performing surgeon. Given the recurrent bouts of abdominal pain with a visualized appendicular orifice and lumen, there was concern about stump appendicitis. The patient subsequently underwent diagnostic laparoscopy when a 3 cm appendicular stump was identified in proximity to the base of the cecum and was subsequently excised.

## Conclusions

Stump appendicitis is a known entity in patients with a prior history of appendectomy who have a residual stump left. Sometimes, it is not picked up on routine imaging like CT. Colonoscopic evaluation of an appendicular orifice can reveal a remnant stump causing a recurrent bout of stump appendicitis.
